# Supplementary material for: Structural modelling and preventive strategy targeting of WSSV hub proteins to combat viral infection in shrimp Penaeus monodon
Source: PLoS One. 2024 Jul 29;19(7):e0307976. doi: 10.1371/journal.pone.0307976 (PMC11285918; doi:10.1371/journal.pone.0307976)
Supplement: S1 File — (PDF) [file pone.0307976.s001.pdf]

Structural Modelling and Preventive Strategy Targeting of WSSV Hub Proteins to Combat Viral Infection in Shrimp *Penaeus monodon*

Tanate Panrat[1,2], Amornrat Phongdara[2], Kitti Wuthisathid[3], Watcharachai Meemetta[3], Kornsunee Phiwsaiya[3,4], Rapeepun Vanichviriyakit[3,5], Saengchan Senapin[3,4], Pakkakul Sangsuriya[6*]

[1]Prince of Songkla University International College, Prince of Songkla University, Hatyai Campus, Songkhla, Thailand

[2]Center for Genomics and Bioinformatics Research, Faculty of Science, Prince of Songkla University, Songkhla, Thailand

[3]Center of Excellence for Shrimp Molecular Biology and Biotechnology (Centex Shrimp), Faculty of Science, Mahidol University, Bangkok, Thailand

[4]National Center for Genetic Engineering and Biotechnology (BIOTEC), National Science and Technology Development Agency (NSTDA), Pathum Thani, Thailand

[5]Department of Anatomy, Faculty of Science, Mahidol University, Bangkok, Thailand

[6]Aquatic Molecular Genetics and Biotechnology Research Team, BIOTEC, NSTDA, Pathum Thani, Thailand

*Corresponding author:

P. Sangsuriya (E-mail: pakkakul.san@biotec.or.th)

Supplementary Figures

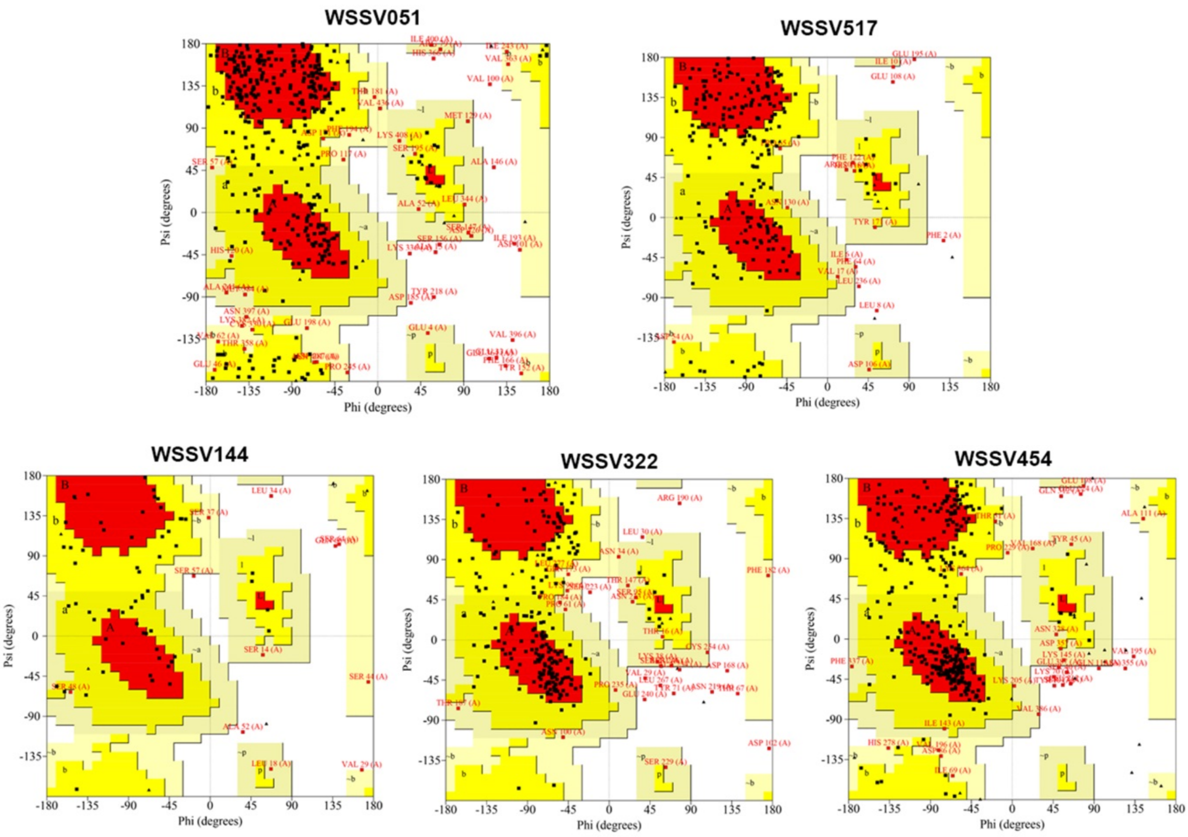

Figure S1. Ramachandran plot analysis of WSSV051, WSSV517 and binding partners. Ramachandran plot analysis on the ProFunc server (http://www.ebi.ac.uk/thornton-srv/databases/ProFunc) was used to assess the quality of the 3D predicted models of WSSV051, WSSV517, WSSV144, WSSV322, and WSV454.

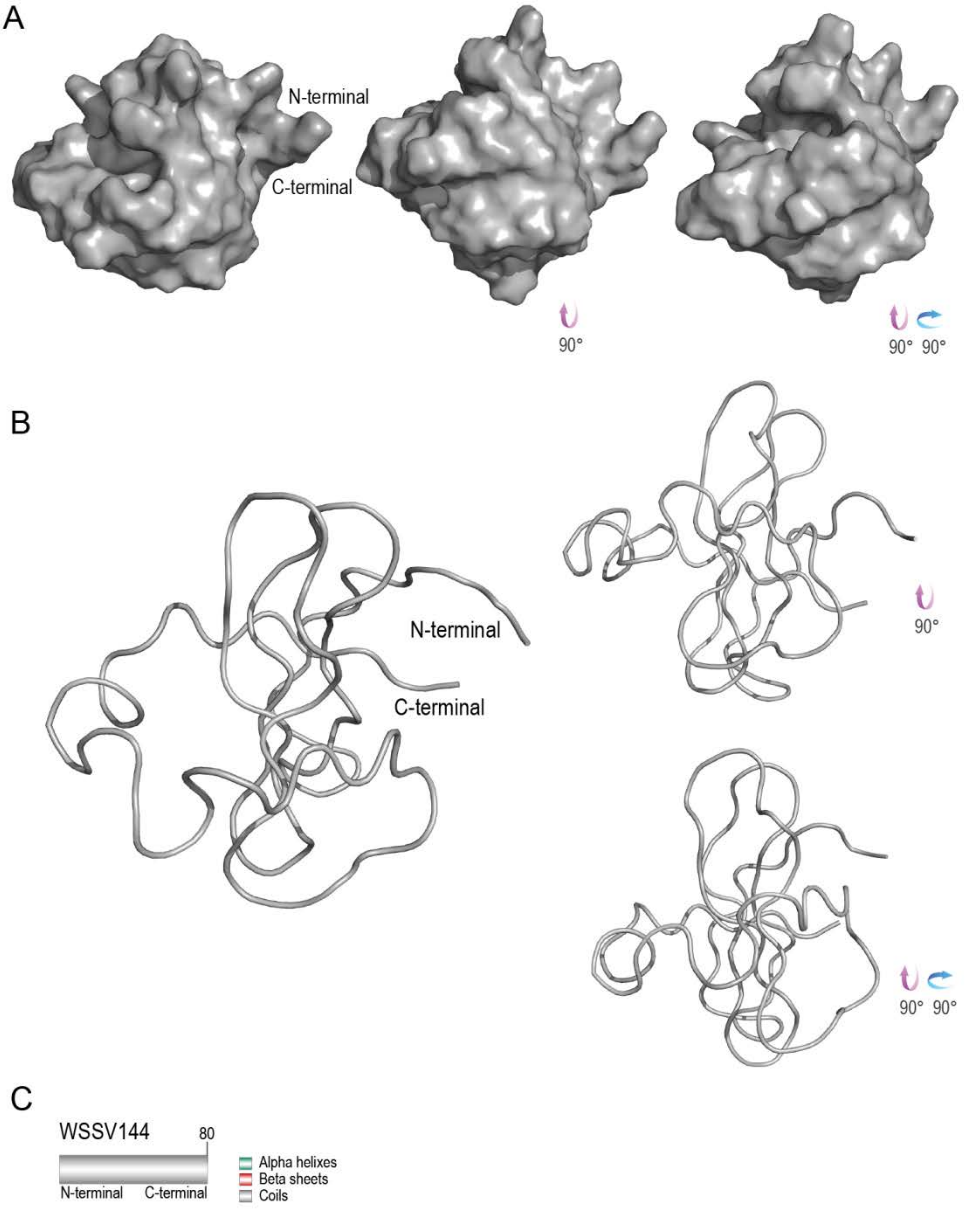

Figure S2. The 3D predicted structure of WSSV144 protein.

(A) The surface structure presented the WSSV144 predicted model. (B) The cartoon structure presented the folding details of WSSV144. (C) The coils segment of WSSV144 colored in pearl. The structural analysis results of WSSV144 suggest that WSSV144 showed in the flexible form and it might facilitate WSSV144 binds with the structure of the target proteins.

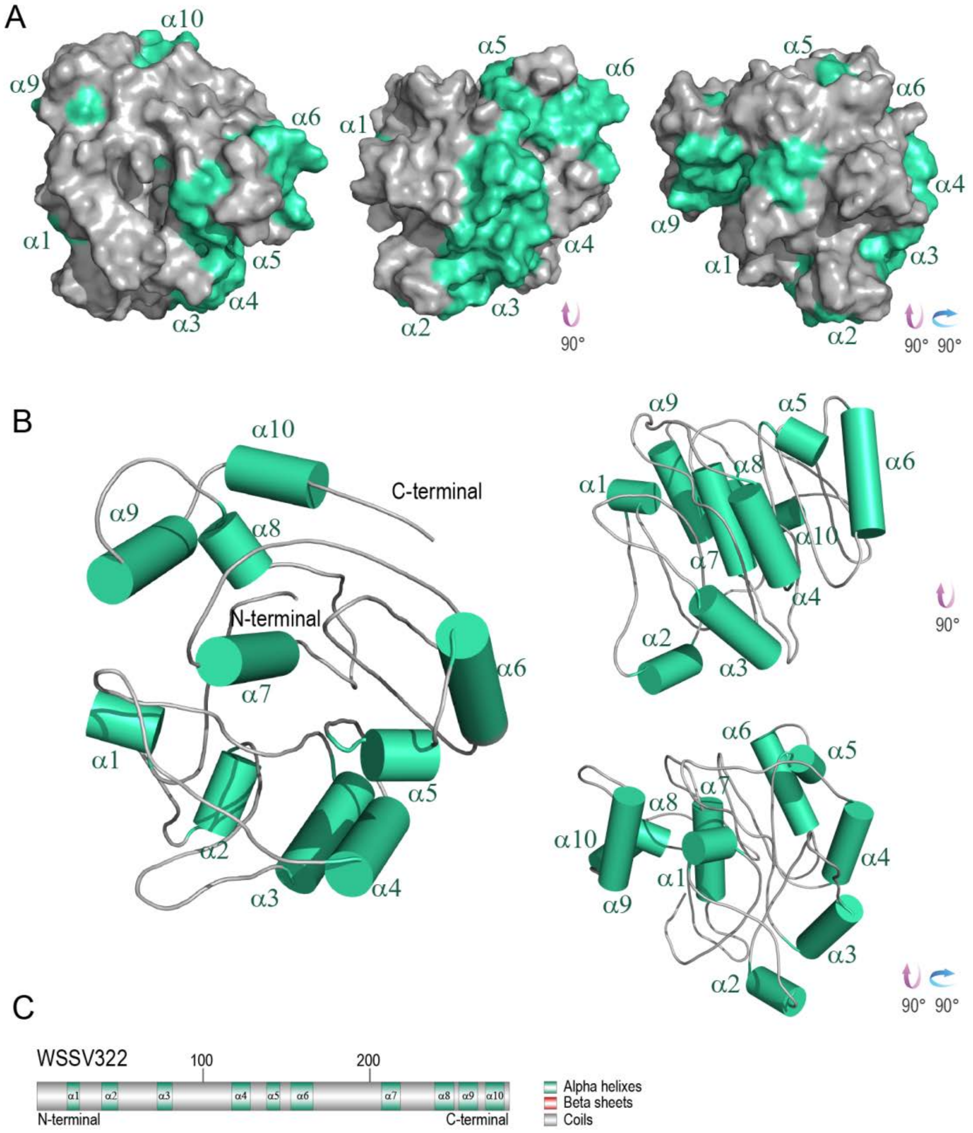

Figure S3. The 3D predicted structure of WSSV322 protein.

(A) The surface structure presented the WSSV322 predicted model. (B) The cartoon structure presented the folding details of WSSV322. (C) The segments colored in turquoise represent α-helices and pearl represent coils. From the structural analysis, we found that the predicted structure of WSSV322 represents only α-helices and coils.

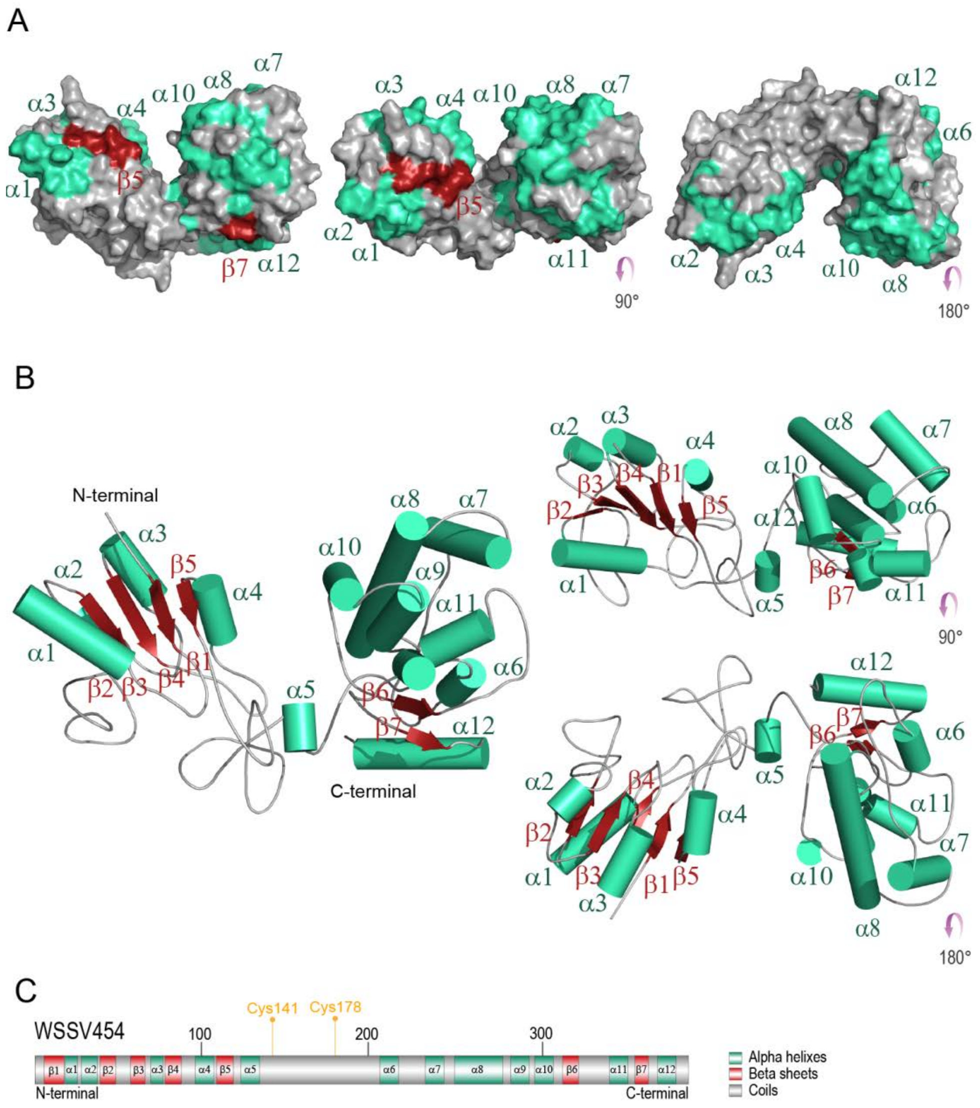

Figure S4. The 3D predicted structure of WSSV454 protein.

(A) The surface structure presented the WSSV454 predicted model. (B) The cartoon structure presented the folding details of WSSV454. (C) The segments colored in turquoise represent α-helices, maroon represents β-sheets, and pearl represent coils. The predicted structure found that the Cys141 and Cys178 formed disulfide-bond that facilities the 3D model of WSSV454 proteins formed the horseshoe shape.

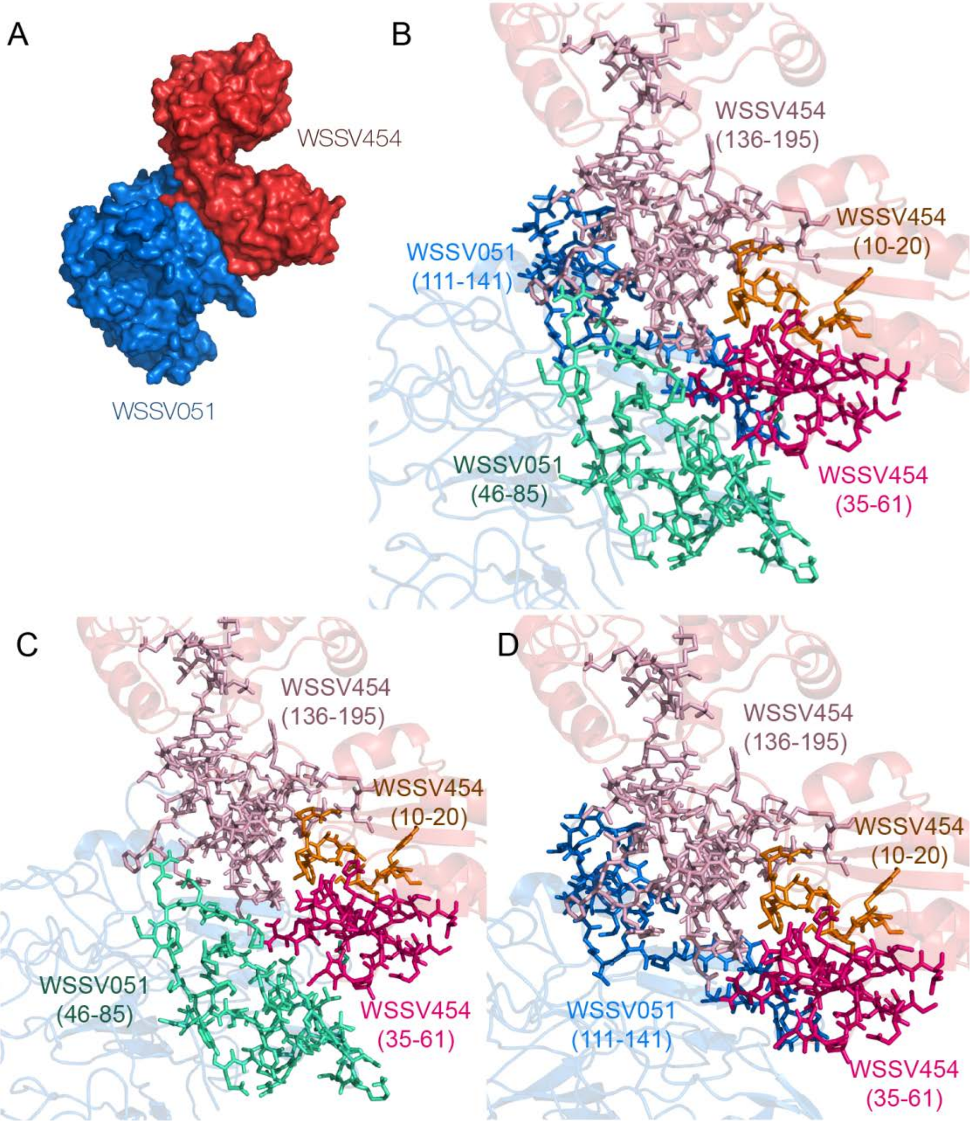

Figure S5. Docking simulation of WSSV051 and WSSV454 interaction Complex.

We performed molecular docking of WSSV051:WSSV454 by using the defaults parameters of the ClusPro 2.0 server and the first ranking of the simulated complex have been selected for explores of the binding sides in details. (A) We assigned 3D model of WSSV051 (colored in marine-blue) as a receptor and WSSV454 (cherry-red) as ligand proteins. (B-D) The stick and bond represent the details of hub protein (WSSV051) interacts with binding protein (WSSV454). The structural analysis of WSSV051:WSSV454 interaction complex found that WSSV454 protein bound to amino acid position 46–85 (green) and 111–141 (blue) of WSSV051 protein with amino acid position 10–20 (orange), 35–61 (red-pink), and 136–195 (light-pink), respectively.

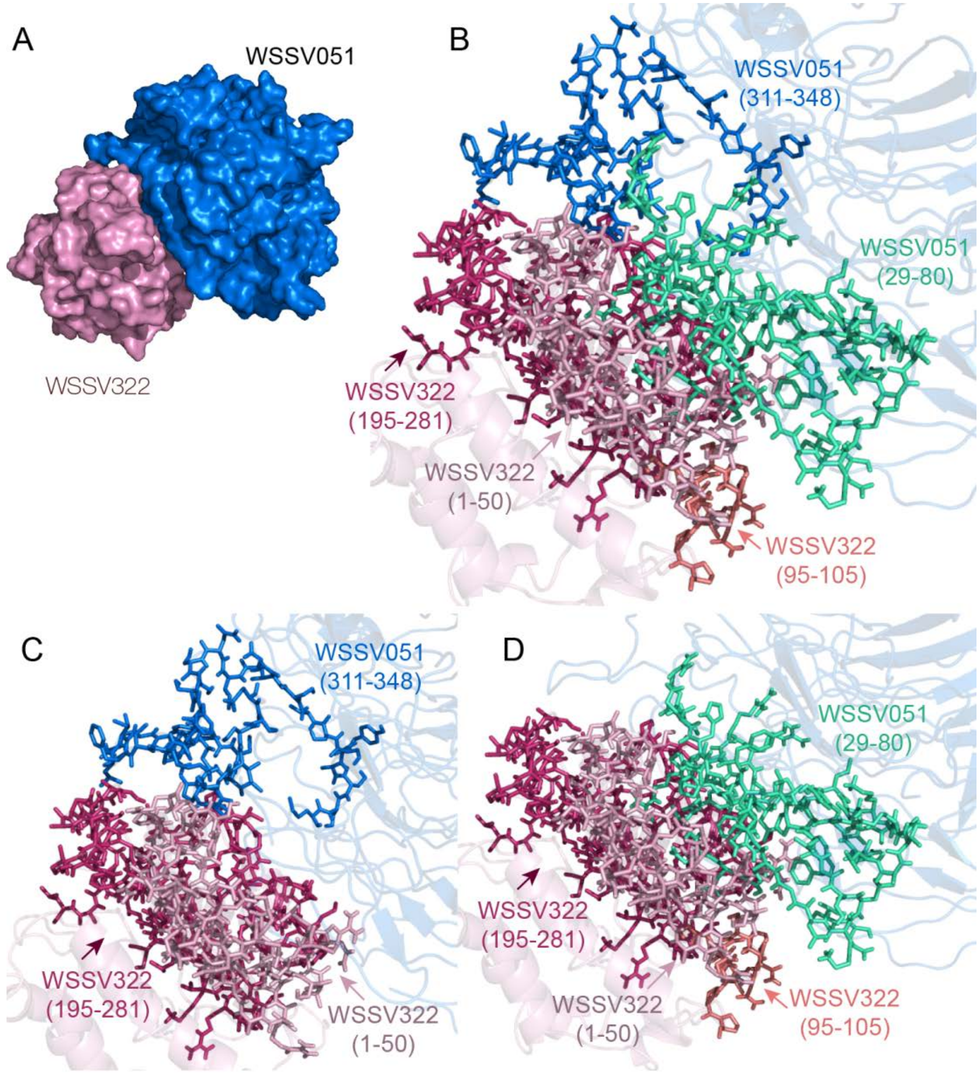

Figure S6. Docking simulation of WSSV051 and WSSV322 interaction complex.

We performed molecular docking of WSSV051:WSSV322 by using ClusPro 2.0 server. (A) We assigned 3D model of WSSV051 (colored in marine-blue) as a receptor and WSSV322 (chocky-pink) as ligand proteins. (B-D) The stick and bond represent the details of hub protein (WSSV051) interacts with binding protein (WSSV322). The structural analysis results of WSSV051:WSSV322 interaction complex found that WSSV322 show the regions at position 1–50 (light-pink), 95–105 (light-orange), and 195–281 (red-pink) bound to WSSV051 at 29–80 (green) and 311–348 (blue), respectively.

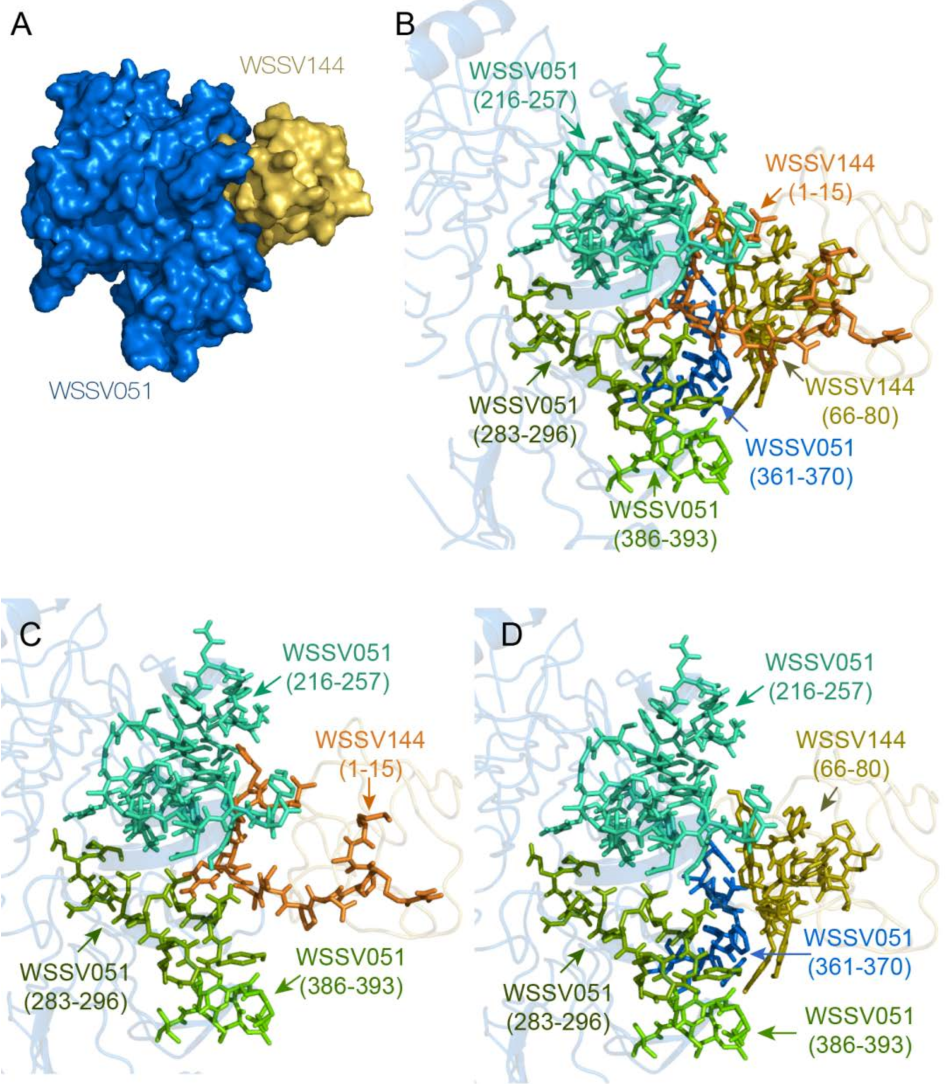

Figure S7. Docking simulation of WSSV051 and WSSV144 interaction complex.

Docking simulation results of WSSV051:WSSV144 interaction complex. (A) The surface structure showed molecular docking complex of WSSV051:WSSV14 whereas the WSSV051 protein (colored in marine-blue) acts as a receptor and WSSV144 (golden-yellow) as ligand proteins. (B-D) The stick and bone represent the details of WSSV051 protein interacts with WSSV144 protein. The analyzed results found that WSSV144 protein interacted WSSV051 at 216–257 (green), 283–296 (lemon-green), 361–370 (blue), and 386–393 (lime-green) with amino acid position 1–15 (orange) and 66–80 (splitpea-green), respectively.

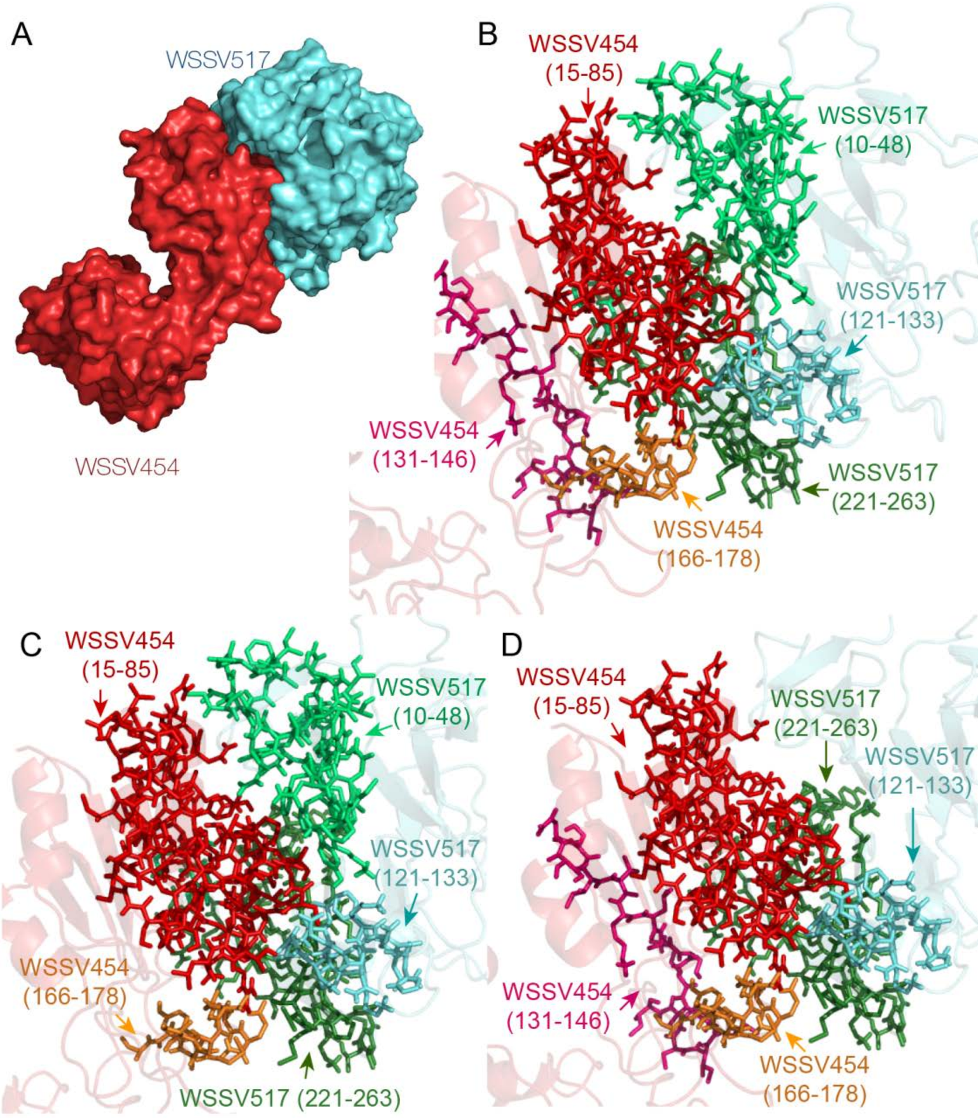

Figure S8. Docking simulation of WSSV517 and WSSV454 interaction complex.

As same as WSSV051:WSSV454, we performed molecular docking of WSSV517:WSSV454 by using the defaults parameters of the ClusPro 2.0 server and the first ranking of the simulated complex have been selected for explores of the binding sides in details. (A) We assigned 3D model of WSSV517 (colored in turquoise) as a receptor and WSSV454 (cherry-red) as ligand proteins. (B-D) The stick and bond represent the details of hub protein (WSSV517) interacts with binding protein (WSSV454). The structural analysis of WSSV517:WSSV454 interaction complex found that WSSV454 protein bound to amino acid position 10–45 (green), 121–133 (blue-green), and 221–263 (dark-green) of WSSV517 protein with amino acid position 15–85 (red), 131–146 (red-pink), and 166–177 (orange), respectively.

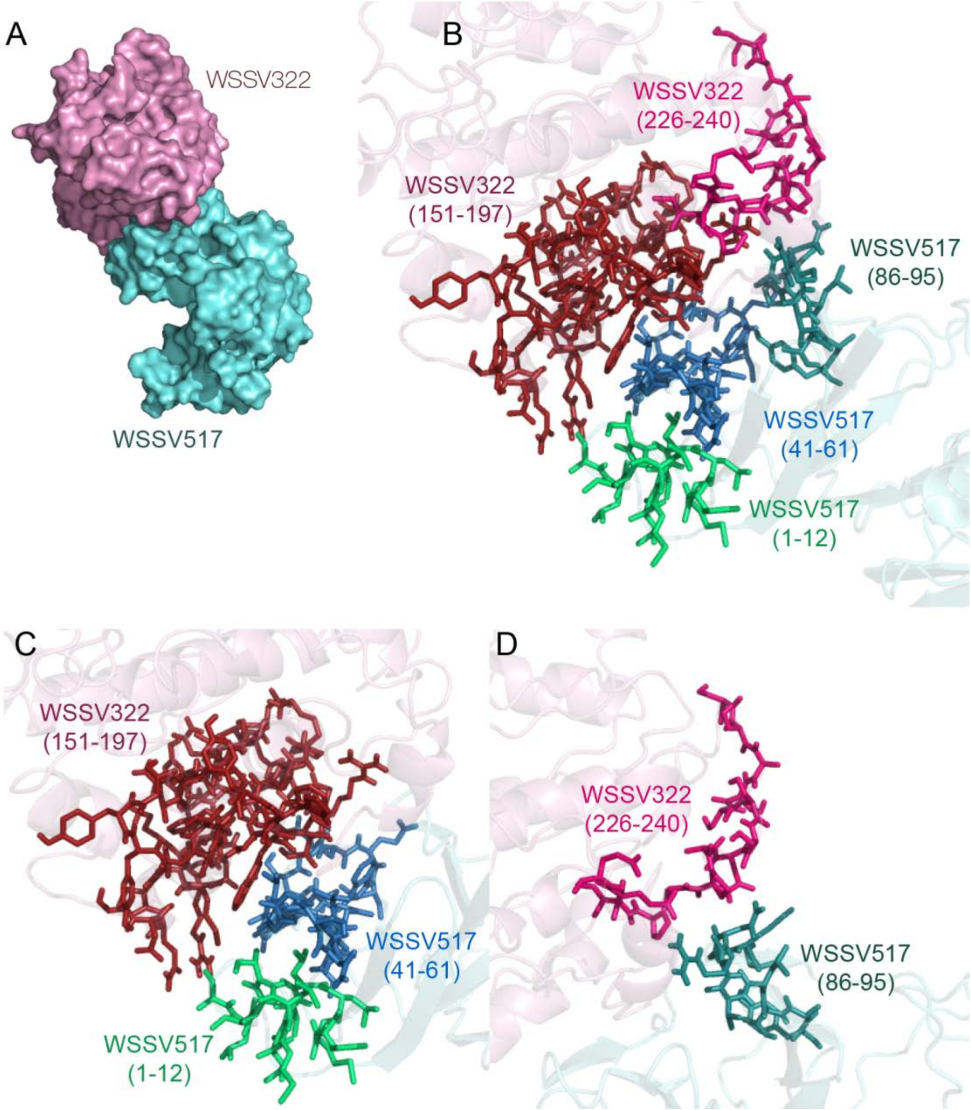

Figure S9. Docking simulation of WSSV517 and WSSV322 interaction complex.

Graphical represent the first ranking of the WSSV517:WSSV322 simulated complex. We assigned 3D model of WSSV517 (turquoise) as a receptor and WSSV322 (chocky-pink) as ligand proteins. (B-D) The stick and bond represent the details of hub protein (WSSV517) interacts with binding protein (WSSV322). The structural analysis results of WSSV517:WSSV322 interaction complex found that WSSV322 show the regions at position 151–197 (red) and 226–240 (red-pink) bound to WSSV517 at 1–12 (green), 41–61 (blue), and 86–95 (blue-green), respectively.

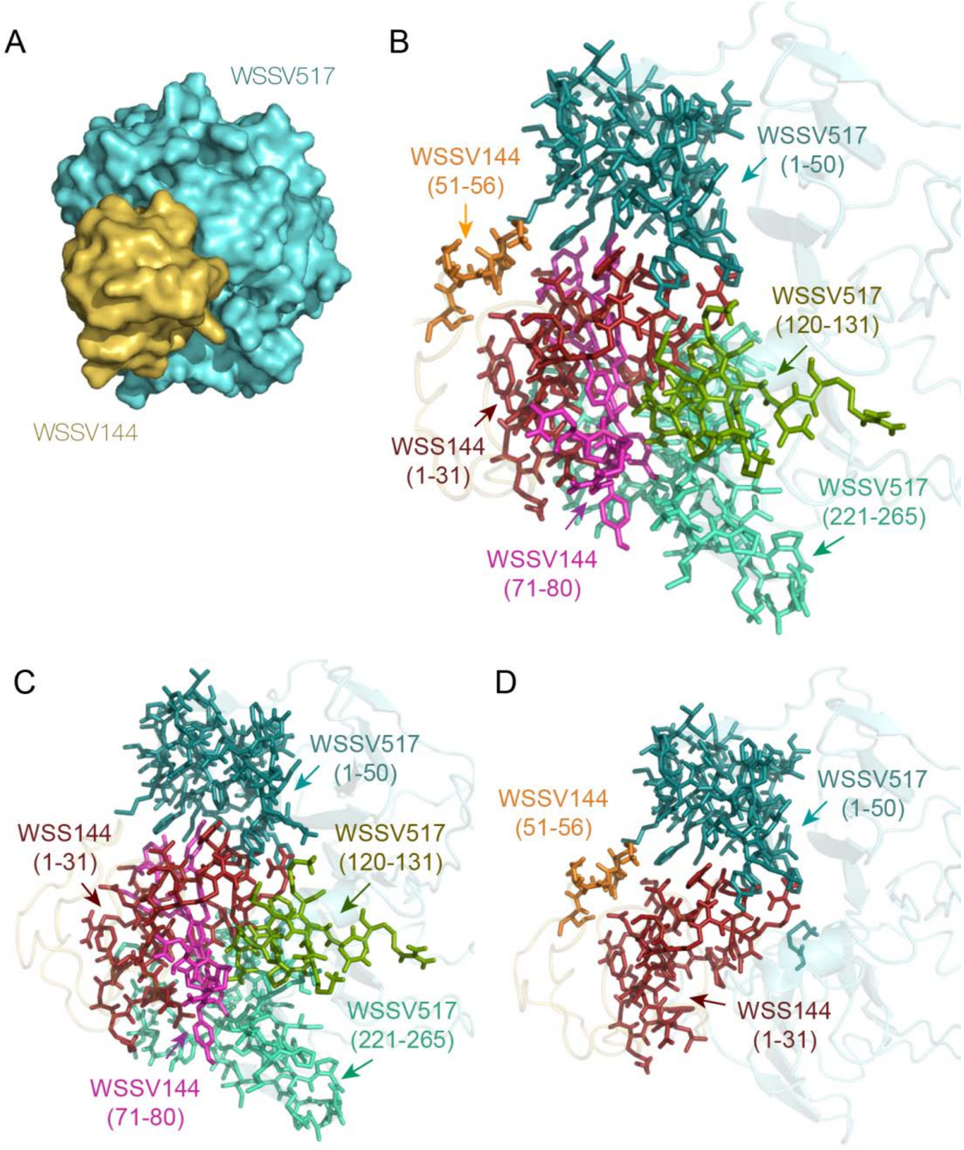

Figure S10. Docking simulation of WSSV517 and WSSV144 interaction complex.

The docking simulation results of WSSV517:WSSV144 interaction complex. (A) The surface structure showed molecular docking complex of WSSV517:WSSV14 whereas the WSSV517 protein (turquoise) acts as a receptor and WSSV144 (golden-yellow) as ligand proteins. (B-D) The stick and bone represent the details of WSSV517 protein interacts with WSSV144 protein. The analyzed results found that WSSV144 protein interacted WSSV517 at 1–50 (blue-green), 120–131 (lemon-green), 221–265 (blue), and 386–393 (lightsea-green) with amino acid position 1–31 (red), 51–56 (orange), and 71–80 (pink), respectively.

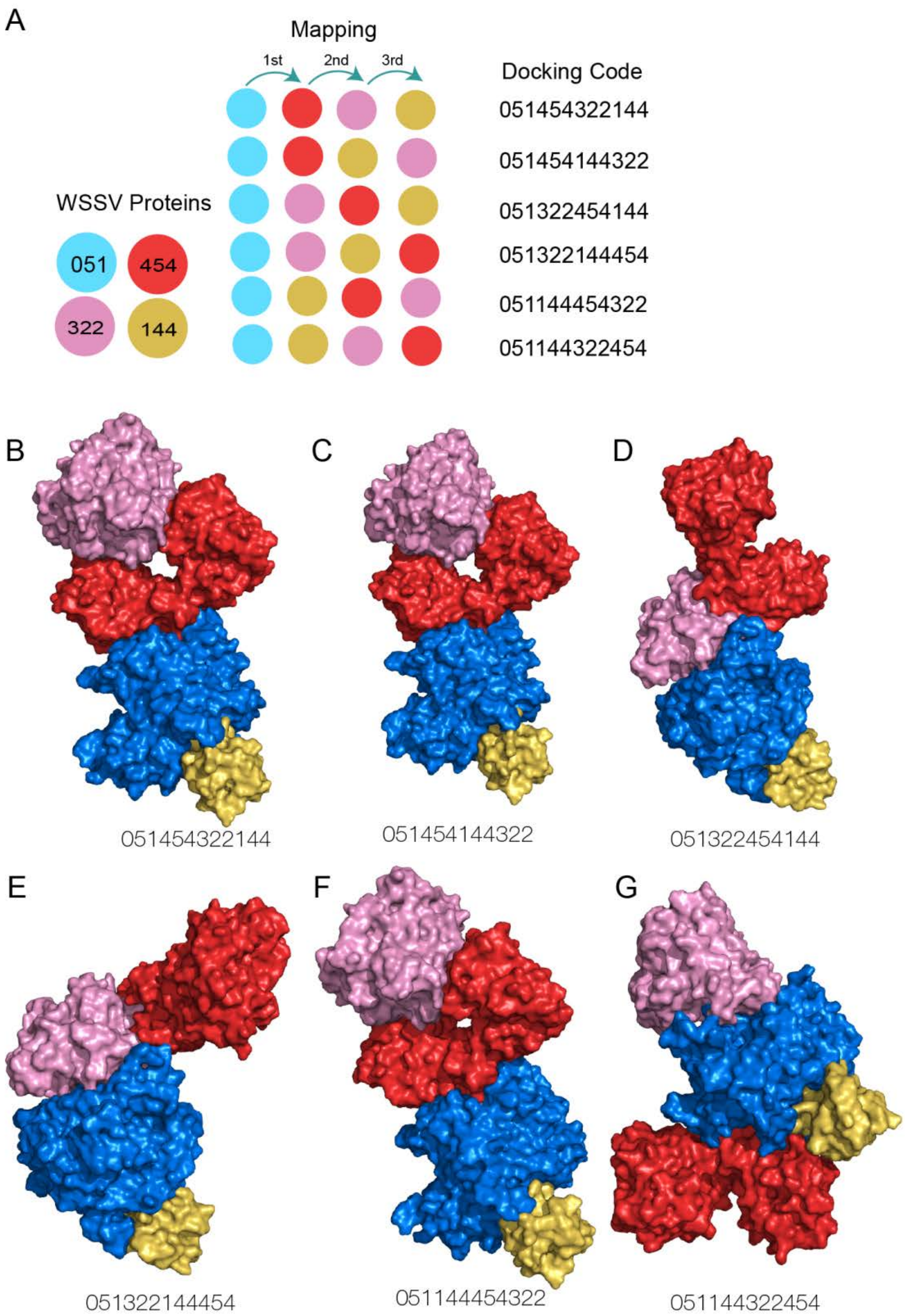

Figure S11. Competitive docking simulation of WSSV051 hub protein with its partners. The graphical represents the competitive structures of possible conformation of WSSV051 interacts with partners. (A) The symbols shown the simulated the modeled of WSSV051 (colored in marine-blue) as a receptor interact with ligand proteins whereas WSSV454 protein colored in cherry-red, WSSV322 in chocky-pink, and WSSV144 in golden-yellow, respectively. (B-G) The illustration shows the competitive structures of the possible conformation of WSSV051 interacts with partners.

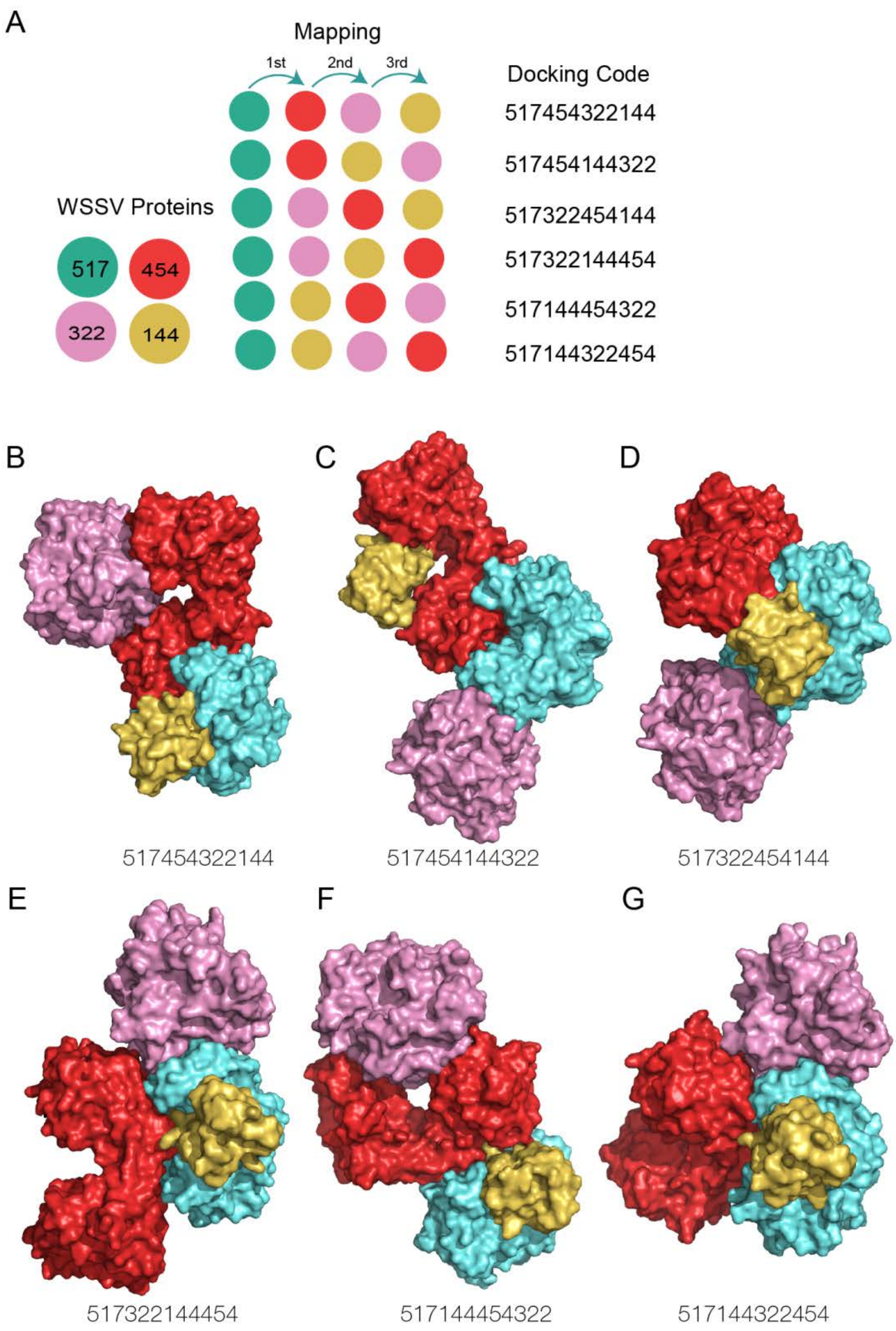

Figure S12. Competitive docking simulation of WSSV517 hub protein with its partners. The graphical represents the competitive structures of possible conformation of WSSV517 interacts with partners. (A) The symbols shown the simulated the modeled of WSSV517 (colored in turquoise) as a receptor interact with ligand proteins whereas WSSV454 protein colored in cherry-red, WSSV322 in chocky-pink, and WSSV144 in golden-yellow, respectively. (B-G) The illustration shows the competitive structures of the possible conformation of WSSV517 interacts with partners.
