# Supplementary material for: Structural modelling and preventive strategy targeting of WSSV hub proteins to combat viral infection in shrimp Penaeus monodon
Source: PLoS One. 2024 Jul 29;19(7):e0307976. doi: 10.1371/journal.pone.0307976 (PMC11285918; doi:10.1371/journal.pone.0307976)
Supplement: S1 Raw images — (PDF) [file pone.0307976.s003.pdf]

Supplementary original gel images

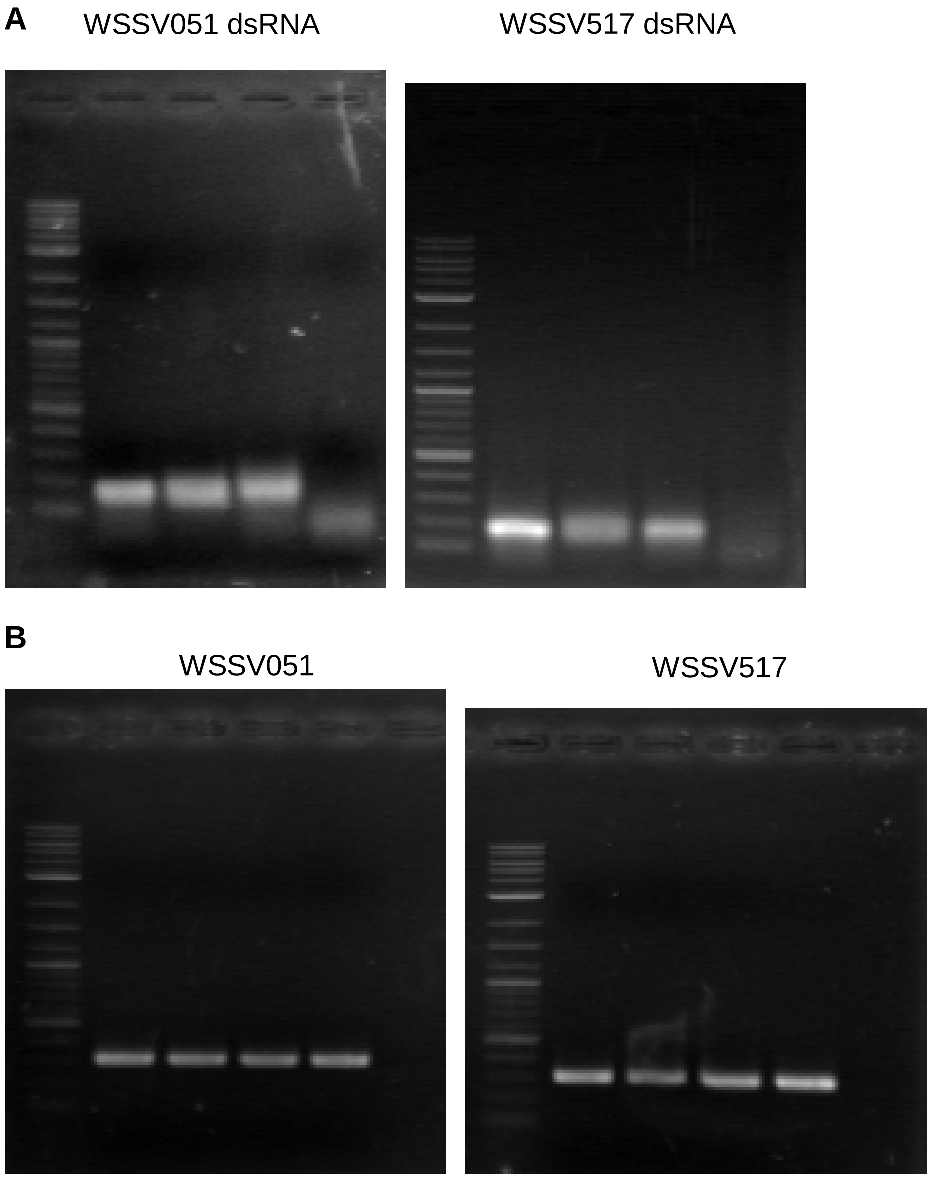

Supplementary gel image 1. The gel images shown in Fig. 6A and 6B were obtained from the Syngene Gel Documentation System.

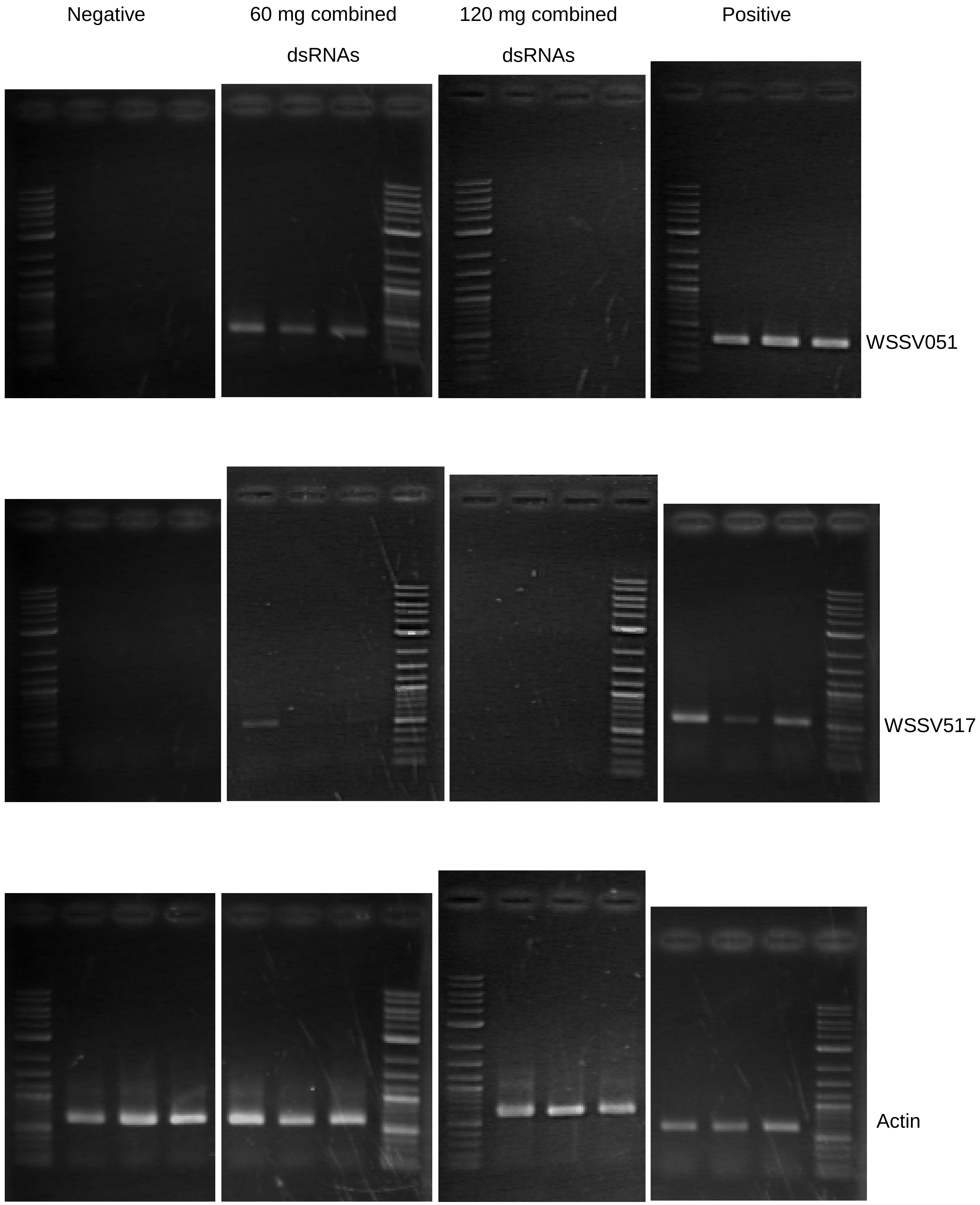

Supplementary gel image 2. The gel images shown in Fig. 8A were obtained from the Syngene Gel Documentation System.

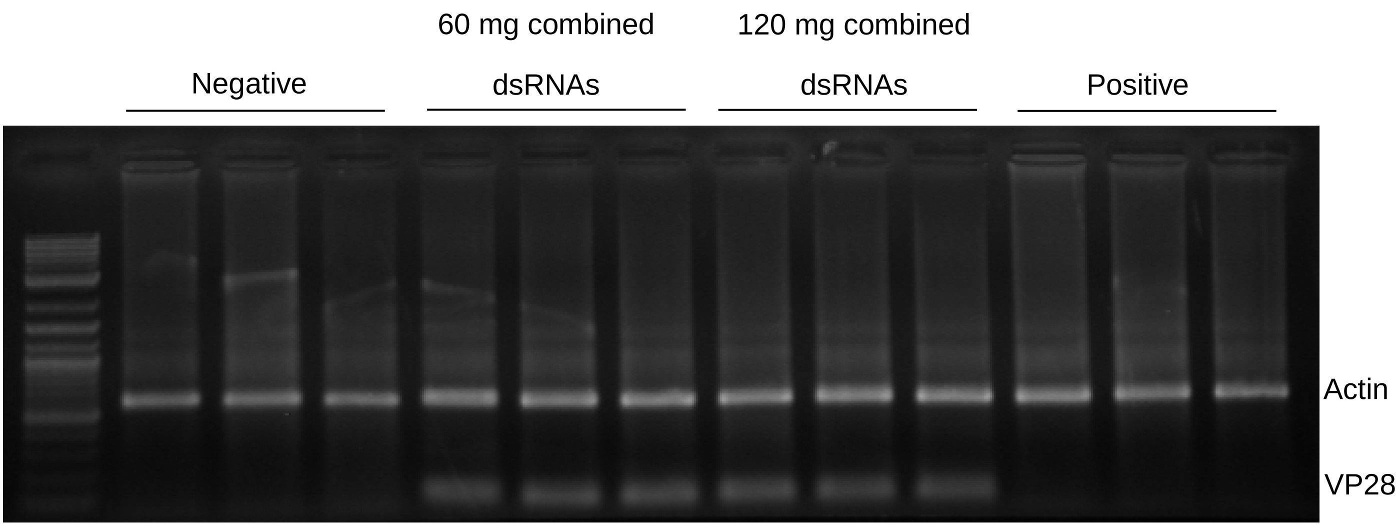

Supplementary gel image 3. The gel image shown in Fig. 8B was obtained from the Syngene Gel Documentation System.
